# COVID-19 Conferences: Resident Perceptions of Online Synchronous Learning Environments

**DOI:** 10.5811/westjem.2020.11.49125

**Published:** 2020-12-14

**Authors:** William Weber, James Ahn

**Affiliations:** The University of Chicago, Section of Emergency Medicine, Chicago, Illinois

## Abstract

**Introduction:**

The coronavirus disease 2019 pandemic forced a rapid transition of in-class residency conferences to online residency conferences; little is known about learners’ perceptions of this new didactic environment. Understanding learners’ perceptions of virtual classrooms can help inform current and future best practices for online, synchronous, graduate medical education.

**Methods:**

We surveyed emergency medicine and internal medicine residency programs at a large urban academic medical center about their perceptions of synchronous online residency conferences.

**Results:**

Residents reported a preference for in-class interactions with peers (85%) and lecturers (80%), with 62% reporting decreased levels of engagement with lecturers during online conferences. Residents reported performing nearly twice as many non-conference-related activities (eg, email, exercise) during online conferences vs in-class conferences. Residents felt that the following methods improved engagement during online conferences: lecturers answering chat questions; small group sessions; and gamification of lectures.

**Conclusion:**

Synchronous online residency conferences were associated with decreased engagement and attention by learners. Simple methods to increase interactivity may help improve the online classroom experience and cultivate novel teaching environments that better support current learning styles.

## INTRODUCTION

Postgraduate physician trainees (residents) complete weekly didactic sessions (conferences) as required by the Accreditation Council for Graduate Medical Education (ACGME).^1^ These conferences traditionally take place in a classroom setting. The SARS-COV-2 coronavirus (COVID-19) pandemic has forced rapid changes to graduate medical education (GME). In the interest of infection control during COVID-19, many residency programs have limited the number of congregating individuals, meaning that in-person lectures have transitioned to synchronous, online learning formats. Such educational environments typically involve learners logging into an online group meeting platform that allows for sharing of presentations as well as discussion over video.

While technology exists for online synchronous resident conferences, such conferences have been common practice for only a small minority of residencies and there is a paucity of research about resident learner perceptions. A meta-analysis of internet-based learning in the health professions in 2008 found similar learner satisfaction and perceived knowledge and skills obtained through online learning; however, less than 10% of the analyzed studies involved synchronous online learning and less than 20% involved residents.^2^ A meta-analysis of instructional design in internet-based learning in health professions was unable to make strong recommendations for practice but identified themes such as interactivity and practice exercises as potential areas of further research.^3^ Many of these themes stem from social learning theory, which has been used as a framework for studying web-based learning environments. Effective web-based learning generally provides learners meaningful interactions with their professors and peers, fosters a culture of learning, and supports multiple learning styles.^4^

Within postgraduate medical education, a recent study of internal medicine program directors found that only 40% of programs had tried synchronous conferences in the past, with less than 20% using synchronous conferences either “often” or “somewhat often.”^5^ The Council of Residency Directors in Emergency Medicine has acknowledged that synchronous online didactics can fulfill educational requirements.^1^ Some residency programs have adopted an asynchronous component to conferences, such as incorporating online learning resources to study, but minimal literature exists on the perceptions of emergency medicine (EM) and internal medicine (IM) residents with synchronous online curricula.

## METHODS

EM and IM residency programs at the University of Chicago were invited to participate in an 11-question survey through established email lists. Survey validity was established by an iterative review process by EM medical education faculty followed by revision and piloting with a representative audience of GME learners from other local institutions. We reviewed feedback from the pilot and incorporated it into the final survey. The survey included an evaluation of learner perceptions of effectiveness of didactic sessions, engagement with lectures, attention to lectures, and performance of simultaneous activities. Residents who completed the survey were invited to enter a drawing for $5 gift cards.

We tabulated and analyzed survey results using Microsoft Excel (Microsoft Corporation, Redmond, WA). The study used two-sample t-tests with an alpha level of <0.01 for significance with a Bonferroni adjustment. For questions with ranges (eg, 0–20% or 1–2 times per hour), we used the average of the range to code the data. The study was approved by the University of Chicago Institutional Review Board: IRB20-0851.

## RESULTS

A total of 81 residents (54% female) participated in the survey: 31 of a possible 49 (63%) EM residents and 50 of a possible 112 (45%) IM residents. The respondents were distributed by postgraduate year (PGY) with 33% PGY 1, 42% PGY 2, and 25% PGY 3 or greater.^5^ The University of Chicago has three-year IM and EM programs, but IM includes a small number of dual-residency IM / pediatrics residents whose training extends further.

**Figure 1:**
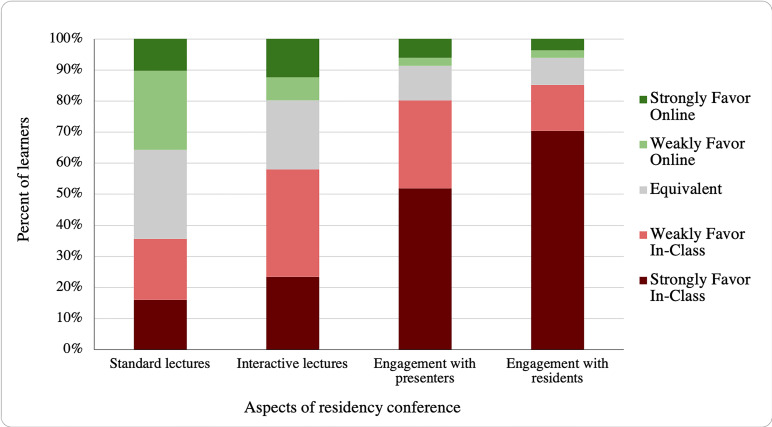
Comparison of perceived effectiveness in aspects of online and in-class residency conferences.

Online conferences had varying degrees of perceived effectiveness. Learners felt that standard lectures translated most easily from an in-class to online format, with a near-even split of those preferring in-class (38%), online (38%), and feeling that the two formats were equivalent in effectiveness (31%) (Figure 1). Learners preferred an in-class setting for interactive lectures, such as game show or question-based formats (58%), with fewer learners preferring online (20%) or feeling equivalence between the settings (22%).

Learners felt that in-class lectures provided more effective engagement with both presenters (80%) and peers (85%) (Figure 1). The majority of learners (62%) reported interacting with presenters less often during online lectures, with a small proportion of students reporting no change (23%) or increased frequency (15%) of interaction during online lectures. In terms of techniques that helped learners engage with a lecturer online, residents felt that a lecturer answering chat questions was most engaging (40%), followed by small-group breakout rooms (21%), and gamification of lectures (audience answering questions for points or prizes) (20%).

Learners reported decreased attention during online conferences (65%) as compared with in-class conferences. When exploring the reasons for decreased attention, learners reported engaging in other activities simultaneously with online conferences. Learners reported a statistically significant increase in frequency of researching conference-related materials, visiting websites not related to conference, reading or composing emails, and completing daily tasks such as exercise or washing dishes (Table 1). In total, learners participated in nearly twice as many non-conference related activities per hour during online conference (4.6 per hour) than during in-class conference (2.4 per hour).

When comparing residency programs on the number and type of simultaneous activities completed during conference, we found no significant differences between EM and IM learners. When given the choice of what percentage of conferences to keep online vs in-class post-COVID-19, residents wanted an average of 42% of conferences online and 58% in-class.

## DISCUSSION

The rapid transition of residency conferences from an in-class to online format during COVID-19 led to a perceived decrease in engagement from learners. While learners felt that lecture-based formats were similar in their effectiveness, they felt that their interactions with both peers and lecturers were not as effective. Social learning theory suggests that peer interaction plays an important role in online learning success.^5^ For instance, attentive classmates model good behavior for a learner and create a positive learning culture. During “screen sharing” presentations, learners may miss body language cues from both the lecturer and their classmates.

Learners suggested multiple techniques that could improve engagement. The top-rated method to increase engagement involved speakers interacting with learners through answering questions from the group chat, consistent with tenets of social learning theory.^4^ A lecturer’s responsiveness to the audience may help participants avoid the feeling of watching a video by allowing them to shape lecture content, consistent with Cook’s suggestion that interactivity promotes learning.^3^ Learners also appreciated small-group breakout sessions and gamification of lectures. All of these techniques support active learning and help overcome the passivity of sitting behind a computer screen.

A majority of learners noted decreased attention with online conferences; this was accompanied by an increase in performing other activities simultaneously with conferences. Residents reported completing nearly twice as many non-conference related activities per hour during online conference than during in-class conference. This increase in potentially distracting activities could lead to residents missing important learning points.

Improving attention could involve structural adjustments as well as a reframing of the conference dynamic. Policies such as keeping webcams enabled could help provide visual feedback to presenters if learners are distracted. The aforementioned methods of creating an interactive synchronous online environment can help focus learner attention. However, an online synchronous classroom setting invites reconsideration of learner behavior. Students in the online environment reported a higher frequency of researching conference-related topics, which provided learners a deeper exploration of conference material in real time. Modifications of the online learning environment to embrace certain benefits of the online format, such as quick breakout rooms, could support diverse learning styles while minimizing unhelpful distractions. While learners reported an increase in simultaneous activities, perhaps not all activities are detrimental to attentiveness. Multiple residents noted that “doing dishes” or “light exercise” allowed them to stay focused for longer periods of time, suggesting that certain simultaneous activities might improve attention for some learners.

## LIMITATIONS

This study took place at a single academic medical center, where local practices or conference formats may differ from other locations. However, the ACGME standards for didactic lectures and the COVID-19 pandemic have forced many institutions to transition to online conference. Response bias is always a possibility, especially when just over half of eligible residents participated in the survey. Finally, the study relied on self-reported attention and effectiveness of learners, which might not accurately capture learner behavior. While the rapidity of COVID-19 social distancing measures prevented pre-pandemic measures of outcomes data, further study could explore learning outcomes and compare current data on online, synchronous conferences with data collected if and when institutions return to in-person conference settings.

## CONCLUSION

The rapid transition from in-class residency conferences to online conferences during the COVID-19 pandemic led to a reported decrease in engagement and attention of learners. Residents also reported increased frequency of multitasking during online conferences, performing nearly twice as many tasks unrelated to conference. Adjustments such as lecturers answering chat questions, small group sessions, and gamification are associated with learners feeling more engaged, aligning with tenets of social learning theory.

## Figures and Tables

**Table 1:** Average frequency of resident learners engaging in other tasks simultaneously with residency conferences.

	In-class mean (times/hour)	Online mean (times/hour)	Mean difference (times/hour), [99% CI]
Researching conference-related materials	0.96	1.45	0.49 [0.35, 0.63]*
Browsing non-conference websites	1.01	1.64	0.63 [0.49, 0.77]*
Emailing	1.05	1.64	0.59 [0.46, 0.72]*
Daily tasks (eg, workout)	0.39	1.40	1.01 [0.90, 1.12]*

* Significant at alpha <0.01. All confidence intervals include a Bonferroni correction.

*CI*, confidence interval.
